# A one health framework to estimate the cost of antimicrobial resistance

**DOI:** 10.1186/s13756-020-00822-6

**Published:** 2020-11-26

**Authors:** Chantal M. Morel, Richard A. Alm, Christine Årdal, Alessandra Bandera, Giacomo M. Bruno, Elena Carrara, Giorgio L. Colombo, Marlieke E. A. de Kraker, Sabiha Essack, Isabel Frost, Bruno Gonzalez-Zorn, Herman Goossens, Luca Guardabassi, Stephan Harbarth, Peter S. Jørgensen, Souha S. Kanj, Tomislav Kostyanev, Ramanan Laxminarayan, Finola Leonard, Gabriel Levy Hara, Marc Mendelson, Malgorzata Mikulska, Nico T. Mutters, Kevin Outterson, Jesus Rodriguez Baňo, Evelina Tacconelli, Luigia Scudeller, the GAP-ON€ network

**Affiliations:** 1 GTGL University of Geneva, Geneva, Switzerland; 2 CARB-X, Boston University, Rockville, USA; 3 Antimicrobial Resistance Centre, Norwegian Institute of Public Health, Oslo, Norway; 4 Infectious Disease Unit, Fondazione IRCCS Ca’ Granda Ospedale Maggiore Policlinico di Milano, Va Francesco Sforza, 28 Milano, Italy; 5 Department of Pathophysiology and Transplantation, University of Milano, Milan, Italy; 6 Department of Management information and production Engineering, Bergamo University, Bergamo, Italy; 7 Drug science Department, Pavia University, Pavia, Italy; 8 Infectious Diseases, Department of Diagnostic and Public Health, Univeristy of Verona, Verona, Italy; 9 CEFAT - Center of Pharmaceuticals Economics and Medical Technologies Evaluation, Drug Science Department, Pavia University, Pavia, Italy; 10 Infection Control Program, Geneva University Hospitals and Faculty of Medicine, Geneva, Switzerland; 11 Antimicrobial Research Unit, College of Health Sciences, University of KwaZulu-Natal, Durban, South Africa; 12 Center for Disease Dynamics, Economics & Policy, New Delhi, India; 13 Antimicrobial Resistance Unit, Department of Animal Health and VISAVET, Complutense University of Madrid, Madrid, Spain; 14 Laboratory Medical Microbiology, University of Antwerp, Antwerp, Belgium; 15 Department of Veterinary and Animal Sciences, University of Copenhagen, Copenhagen, Denmark; 16 Division of Infectious Diseases, Geneva University Hospitals and Faculty of Medicine, Geneva, Switzerland; 17 Global Economic Dynamics and the Biosphere, Royal Swedish Academy of Sciences, Stockholm Resilience Centre, Stockholm, Sweden; 18 Stockholm Resilience Centre, Stockholm University, Stockholm, Sweden; 19 Division of Infectious Diseases, Infection Control and prevention program, Antimicrobial Stewardship program, AUBMC, Beirut, Lebanon; 20 Veterinary Pathobiology, School of Veterinary Medicine, University College Dublin, Dublin, Ireland; 21 Infectious Diseases Unit, Hospital Durand, Buenos Aires, Argentina; 22 Infectious Diseases and Clinical Microbiology, Universidad Maimónides Buenos Aires, Buenos Aires, Argentina; 23 Division of Infectious Diseases & HIV Medicine, Department of Medicine, Groote Schuur Hospital, University of Cape Town, Cape Town, South Africa; 24 Division of Infectious Diseases, University of Genova and San Martino Hospital, Genova, Italy; 25 Bonn University Hospital, Institute for Hygiene and Public Health, Bonn, Germany; 26 Infectious Diseases Division, Hospital Universitario Virgen Macarena, Sevilla, Spain; 27 Department of Medicine, University of Seville / Biomedicine Institute of Seville, Sevilla, Spain; 28 Internal Medicine I, Tübingen University, Tübingen, Germany; 29 IRCCS Policlinico San Matteo Foundation, Pavia, Italy

**Keywords:** Antimicrobial resistance, Cost, One health

## Abstract

**Objectives/purpose:**

The costs attributable to antimicrobial resistance (AMR) remain theoretical and largely unspecified. Current figures fail to capture the full health and economic burden caused by AMR across human, animal, and environmental health; historically many studies have considered only direct costs associated with human infection from a hospital perspective, primarily from high-income countries. The Global Antimicrobial Resistance Platform for ONE-Burden Estimates (GAP-ON€) network has developed a framework to help guide AMR costing exercises in any part of the world as a first step towards more comprehensive analyses for comparing AMR interventions at the local level as well as more harmonized analyses for quantifying the full economic burden attributable to AMR at the global level.

**Methods:**

GAP-ON€ (funded under the JPIAMR 8th call (Virtual Research Institute) is composed of 19 international networks and institutions active in the field of AMR. For this project, the Network operated by means of Delphi rounds, teleconferences and face-to-face meetings. The resulting costing framework takes a bottom-up approach to incorporate all relevant costs imposed by an AMR bacterial microbe in a patient, in an animal, or in the environment up through to the societal level.

**Results:**

The framework itemizes the epidemiological data as well as the direct and indirect cost components needed to build a realistic cost picture for AMR. While the framework lists a large number of relevant pathogens for which this framework could be used to explore the costs, the framework is sufficiently generic to facilitate the costing of other resistant pathogens, including those of other aetiologies.

**Conclusion:**

In order to conduct cost-effectiveness analyses to choose amongst different AMR-related interventions at local level, the costing of AMR should be done according to local epidemiological priorities and local health service norms. Yet the use of a common framework across settings allows for the results of such studies to contribute to cumulative estimates that can serve as the basis of broader policy decisions at the international level such as how to steer R&D funding and how to prioritize AMR amongst other issues. Indeed, it is only by building a realistic cost picture that we can make informed decisions on how best to tackle major health threats.

## Background

### AMR costs - the limitation of current macro and micro estimates

In recent years, numerous global institutions including the United Nations [1], World Health Organization (WHO) [2], EU commission [3, 4], World Bank [5], Food and Agriculture Organization of the United Nations (FAO) [6], Organization for Economic Cooperation and Development (OECD) [7, 8], as well as national governments such as that of the United Kingdom [9, 10] have acknowledged the importance of quantifying the costs of antimicrobial resistance (AMR).

While older studies focussed on costs to health services, more recent studies have included many other costs that AMR imposes on society more widely. These costs derive from the prolongation of illness and increased levels of mortality, work absenteeism and reduced labour efficiency, disruption in international trade, reduced livestock production [11].

The more publicised estimates of the cost of AMR have been at the macro-economic level, with forecasts far into the future, using extrapolation and projection from mathematical modelling. The results of these studies have been staggering. The impact of AMR over the years until 2050 is estimated by the World Bank to be within the same order of magnitude as that of the major 2008 global financial crisis [5]: even in the optimistic low-AMR scenario, the simulated losses of world output exceed $1 trillion annually after 2030 and reach $2 trillion annually by 2050 [9]. The estimated loss in economic output attributable to AMR, if no interventions are implemented, will be 0.14% of global GDP every year. Overall, AMR threatens to rollback economic and food security gains made over the past 50 years [6] with developing countries being disproportionately more affected [10]. Sustainable development goals relating to poverty, childhood survival, and development could be jeopardised, with an additional 28 million people estimated to fall into extreme poverty by 2050 compared to 2017 [5].

While useful for giving a sense of the scale and high cost of inaction globally, these are rough, top-down estimates, based on numerous parameters with significant data gaps and thus requiring many assumptions. Such estimates are of limited use for understanding the full cost of AMR at the local level or for making decisions about how to tackle AMR in an economically optimal manner. The nature of the current data also hinders our ability to make appropriate trade-offs between policy options. Where estimates have been made on a micro level, they have looked mainly at costs derived from the human health burden imposed by AMR, ignoring indirect costs and costs derived from resistance in animals and the environment [2]. This again limits the use of such estimates to make comparisons amongst potential policy and practice interventions and to inform policy.

The GAP-ON€ network is funded through the Joint Programming Initiative on Antimicrobial Resistance (JPIAMR) 8th call as part of the Virtual Research Initiative 2018 [12]. Its purpose is to bring together experts in the AMR field in order to identify essential AMR costs -- taking a global, One Health perspective -- that could be used to conduct economic analyses bottom-up, to inform local decisions, and to build a more nuanced overall cumulative cost picture.

## Methods

### Expert consultation

Expert consultation took place across the GAP-ON€ network, which includes 19 smaller networks and institutions, comprising human and veterinary infectious diseases physicians and microbiologists, experts in AMR burden estimation, food safety, health-economics, international law, as well as infection control experts, clinical epidemiologists, statisticians, and health information librarians.

The costing framework was constructed by means of teleconferences, Delphi rounds and a face-to-face meeting, to reach consensus on the perspective of the framework, the list of relevant pathogens, applicable costs, epidemiological data requirements, and data quality dimensions. Interim reports and feedback were managed through electronic exchange. A REDCap survey tool [13] was used to quantify views on key issues. Decisions were made using consensus methods. At each step, consensus was defined as > 80% of the panel agreeing with < 10% disagreeing. Where consensus was not reached initially, refinements and discussion continued until a qualifying level of agreement could be reached. Overall, two teleconferences were held (June and September 2019) before the face-to-face meeting (Verona, Italy, 24–25 September 2019), followed by the 4 Delphi rounds, and one more TC (January 2020).

### Definition of AMR

The focus of this work is currently limited to bacterial resistance to antibiotics. We maintain the term “antimicrobial resistance” to highlight that this work can later be extended to antifungal, antiviral and antiparasitic resistance. The framework focusses on the costs associated with colonization or infection by single or multiple resistant pathogens, as opposed to its antibacterial sensitive counterpart.^1^

For the purposes of this costing exercise, colonization was defined as the presence of bacteria from a non-sterile specimen (nasal swabs, urine, skin, sputum etc.) in a sufficiently high concentration that it can be detected, but without causing signs or symptoms of disease. Colonization can persist for days to years, and a person or animal colonized with a drug-resistant organism may transmit it to other humans/animals; it may not always impose additional costs.

Infection was defined as clinically and (ideally) microbiologically confirmed disease, including identification of an invasive pathogen from a specimen combined with infection-specific symptoms; it generally imposes costs within both human and animal health settings at some level.

### Selection of most relevant pathogen-drug pairs

The starting point for the selection process was the WHO list of priority antibiotic-resistant bacteria [14]. In addition to this list, all bacteria resistant to drugs believed to be most relevant for human, animal, and plant health were added by consensus methods as described above.

### Selection of sectors

**Fig. 1:**
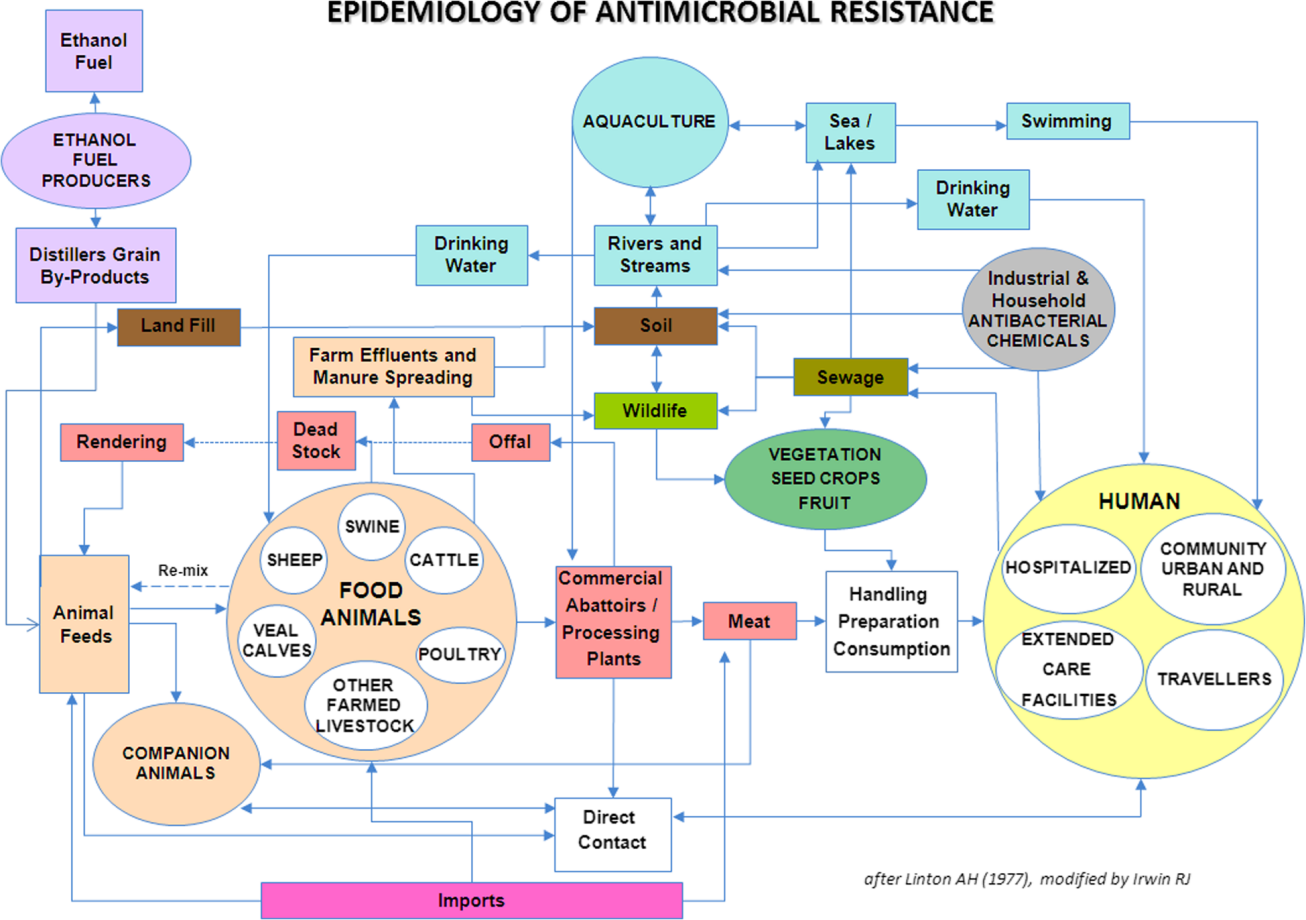
Settings in which antibiotics are used and potential for transmission of resistance bacteria (and resistance genes) [15]

This work includes all of primary setting in which antibiotics are used, and where there is potential for transmission of resistant bacteria (and resistance genes) [15] (Fig. 1).

## Results

**Fig. 2:**
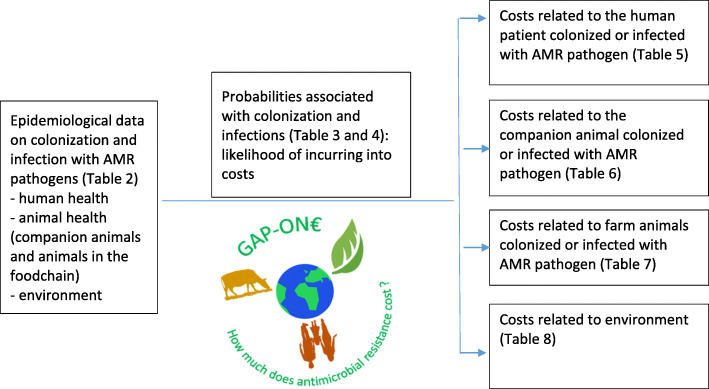
the GAP-ON€ framework

The resulting framework consists of the epidemiological data and the cost components that need to be collected to build a credible picture of AMR-costs from the local level (Fig. 2).

### Epidemiological data components

AMR pathogens can cause either colonization or disease in human or animal health, and can be transmitted within and between One Health areas (Table 1).

**Table 1:**
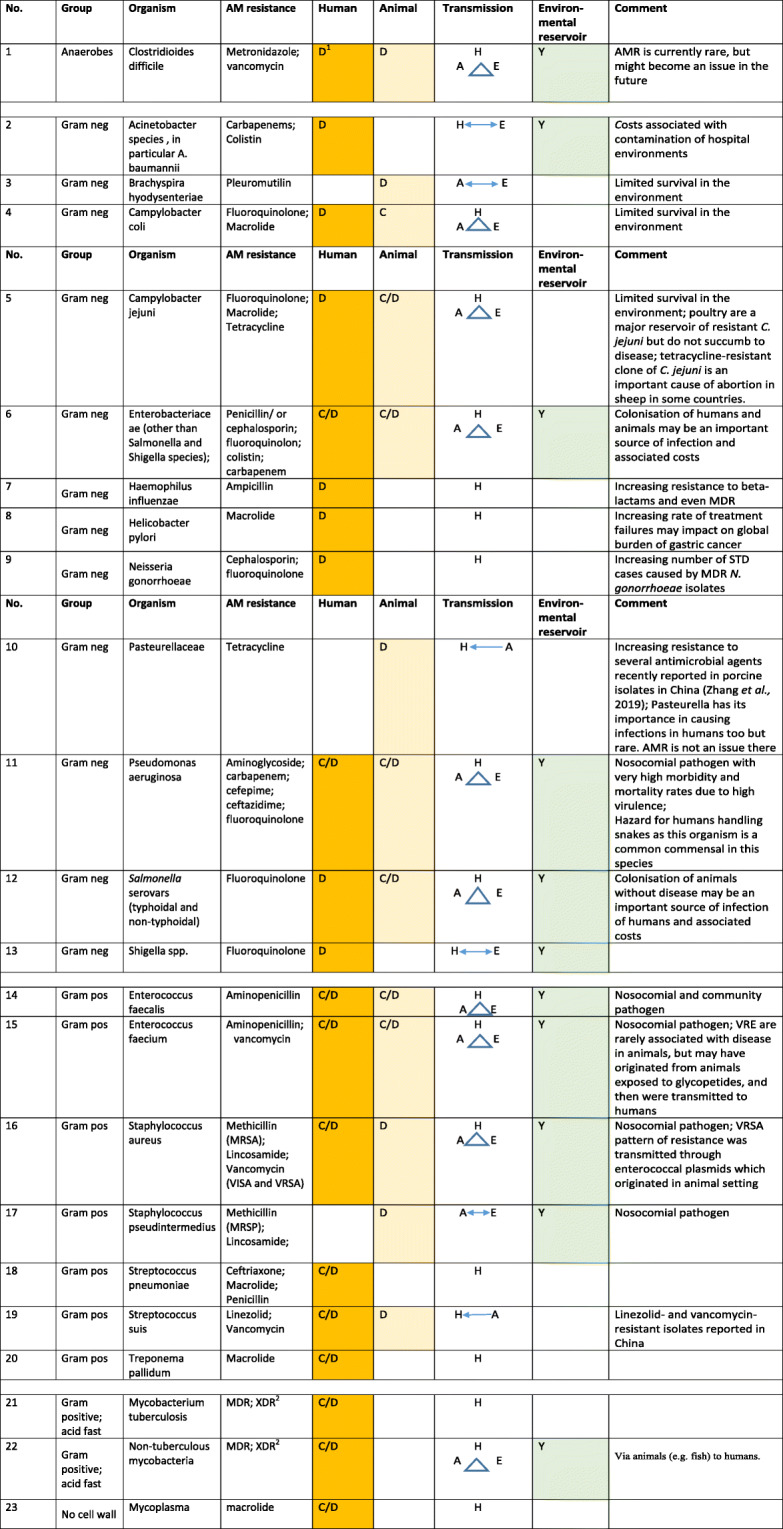
Antimicrobial resistant bacteria with cost implications in human health or in animal health and production

Important routes of transmission and the role of the environment as a reservoir of infection are indicated

^1^D = disease occurs due to the organism(s), C = no disease but costs associated with colonisation; H = human, A = animal, E = environment, Y = yes

^2^In the context of mycobacterial infection, MDR is defined as resistance to at least isoniazid and rifampin and XDR as resistance to isoniazid and rifampin and at least 3 of the 6 classes of aminoglycosides, polypeptides, fluoroquinolones, thioamides, cycloserine, and para-aminosalicyclic acid

Probabilities can be estimated based on the epidemiological data collected (Tables 2, 3 and 4). Incidence and prevalence are both important, as are both colonisation and infection, and the probability of colonisation leading to infection. These data should be derived from proper surveillance studies or from other relevant study designs (e.g. longitudinal studies to estimate probability of transition from colonised to infected status) [18]. If available, estimates surrounding the potential for transmission across settings and transmission rates within settings (human to human or animal to animal transmission) should also be considered (see Fig. 1).

**Table 2:** Epidemiological data necessary to build a “One-Health” cost model of AMR

Data element	Data items
Prevalence of colonisation (Human Health)	Proportion of individuals colonised by drug-resistant pathogens, by subgroups (please see text) at the time of health care access Proportion of individuals colonised by drug-resistant pathogens, by subgroups at point prevalence studies
Prevalence of colonisation (Animal Health)	Proportion of food animals colonised by drug-resistant pathogens post-slaughter
Prevalence of colonisation (Environment)	Presence/absence of drug-resistant pathogens in water, soil and air Relevant to Human health: • Proportion of healthcare facility or home surfaces contaminated with resistant microorganisms (implies choice of relevant surfaces) • Abundance and diversity of drug-resistant pathogens in health facility/household effluent Relevant to Animal health: • Proportion of animal housing, abattoir and food preparation surfaces contaminated with resistant microorganisms (implies choice of relevant surfaces) • Abundance and diversity of drug-resistant pathogens in farming, abattoir and food preparation entities (restaurants & food processing plants) effluent The Environment: • Abundance and diversity of drug-resistant pathogens per volume/quantity of water, soil or air measured.
Prevalence of infection (Human Health)	Number of patients with infection, out of overall number of patients in the health care setting in that specific subgroup, at the time of assessment
Prevalence of infection (Animal Health)	Number of animals with infection, out of overall number of animals in the veterinary care/farm setting in that specific subgroup, at the time of assessment
Incidence of colonisation (Human Health)	Number of new colonisations over an appropriate denominator (implies choice of denominator: see Table 2 in [16]) e.g. in outbreaks of KPC in NICUs, the number of new colonisations is also taken into account [17] For example, number of unique cases of colonised *Clostridium difficile* cases identified over 1000 patient-days (i.e. incident rate)
Incidence of colonisation (Animal Health)	Number of new colonisations out of animals not colonised
Incidence of infection (Human Health)	Number of patients with new infection caused by a pathogen resistant to 1st line, 2nd line, 3rd line antimicrobials, or MDR, by an appropriate denominator (implies choice of denominator: see Table 2 in [16]), by subgroup
Incidence of infection (Animal Health)	Number of animals with new infection caused by a pathogen resistant to 1st line, 2nd line, 3rd line antimicrobials, or MDR, by an appropriate rate denominator (implies choice of denominator), by subgroup

**Table 3:** Probabilities associated with colonisation necessary in a “One-Health” cost model

Data element	Probability
Morbidity (Human health)	Probability of developing infection in colonised individuals, Probability of contact precautions/isolation when colonised Probability of lower quality care when colonised or of missed care opportunity (e.g., surgical prophylaxis not administered in patients known to be colonised) Probability of undergoing diagnostic tests Probability of non-standard surgical prophylaxis Probability of being treated even in absence of infection, due to known colonisation status (increase in selection pressure related to environmental contamination) Lower quality of life
Morbidity (Animal health)	For companion animals, probability of being screened for colonisation with AMR pathogens (e.g. MRSA) in referral veterinary practices. Probability of developing infection in colonised animals Probability of surveillance of faecal samples for MDR organisms under public health programmes
Mortality (Animal health)	Probability of the animal being slaughtered due to colonisation with resistant pathogen.
Screening (Humans / animals)	Probability of starting a screening programme when there is a colonised patient (Number of colonised patients to trigger a screening programme) (humans / animals)
Bi-directional Transmission of colonisation between One Health Areas	Probability of each of 6 possible broad paths between One Health areas, and within-area probability of transmission (e.g. between LTCF and hospitals and vice versa)

**Table 4:** Probabilities associated with infection necessary in a “One-Health” cost model

Data element	Probability
Mortality (overall) (Human and animal health)	Probability of dying **WITH** a MDR infection, for a patient/animal infected (by the subgroups defined in “epidemiology”) Treatment efficacy of 2nd line, 3rd line etc. drugs
Mortality (attributable) (Human and animal health)	Probability of dying **FROM** an MDR infection, for a patient/animal infected (by the subgroups defined in “epidemiology”) Treatment efficacy of 2nd line, 3rd line etc. drugs
Morbidity (Human and animal health)	Probability of developing long term consequences (e.g chronic or recurrent infections, long term disability from ICU stay, lower QoL, etc) from AMR infections, out of all patients infected Probability of developing adverse events, if treated with 2nd, 3rd etc. line drugs Longer hospital stay in patients/animals with AMR infections, compared to those with the same, but not AMR, infection Longer ICU stay for patients/animals with AMR infections
Additional diagnostic procedures for drug resistant infections (Human and animal health)	Probability of undergoing additional diagnostic procedures (e.g. imaging to diagnose site of infection or foci of distant infectious metastatic foci, FollowupFollow-up blood cultures, etc)
Screening (Humans / animals)	Probability of starting a screening programme when there is an infected patient/animal (Number of colonised patients to trigger a screening programme) (humans / animals)
Insurance	Probability of having an insurance to cover extra AMR costs (Please note that pet insurance is rare in most countries)

#### Transmission of resistance between pathogens

The literature confirms the presence of similar strain types, clones, resistance genes and associated mobile genetic elements across human, animal and environmental health in different permutations and combinations where two or more elements of the One Health triad have been assessed [19]. However, transmission dynamics and directionality have yet to be ascertained and some assumptions may have to be made if such estimates are still unavailable at the time of the cost evaluation exercise.

### Adapting the cost framework to the antibiotic resistance scenario

The costs associated with resistance depend on the antibiotic to which resistance has developed as well as other contextual factors. Some potential scenarios include the following:

1. Single drug resistance

Resistance emergence to a drug that was previously effective and widely used, but where there are equally effective and safe alternatives that are readily available; in this case the costs will mainly revolve around more expensive treatment, more diagnostics, more frequent side-effects/lower tolerability, and longer hospital admission (i.e. mainly health care costs and R&D costs).

2. Multi-drug resistance

Resistance emergence to drugs, where equally effective and safe alternatives are not readily available, but the alternative drug(s) becomes more readily available as transmission increases; here the costs need to cover all of the above, plus those associated with worsening infection, possibly more often resulting in death, acquiring a more expensive treatment, side-effects/lower tolerability (i.e. health care costs, some individual productivity loss, and R&D costs).

3. Pan-drug resistance

Resistance emergence to a last resort antibiotic (creating a pan-resistant pathogen); here opportunity costs with regards to avoiding high-risk treatments should be considered, the costs/burden beyond the health sector will become more pertinent, like lost productivity and trade, less tourism, expedited R&D, etc. (i.e. assume progressive economic lockdown beginning at an effective reproduction number > 1, as seen in the current COVID-19 crisis).

### Cost data summary (see tables for more detailed cost itemisation)

#### Cost burden of AMR on humans (Table 5)

Direct costs associated with care in each of the relevant health care settings should be considered (e.g. long-term care facilities, outpatient visits, inpatient stays, intensive care, etc.). Indirect costs deriving from the time away from work for patients and carers, informal care by others and their loss of productivity, should also be included. Costs related to healthcare avoidance should also be considered. Cumulatively, any loss in productivity due to absence or disabilities related to resistant infection should also be included.

**Table 5:** Costs related to the patient colonised or infected with resistant pathogen in human health, necessary in a “One-Health” cost model

Data element	Category of costs	Cost items
Direct costs	Costs of any treatment or prophylaxis of the patient borne by the health service (regardless of whether or not such costs are passed on to the payor/insurance company).^a^	− Cost of antibiotics for treating infections − Higher antibiotic expenses for empirical therapy due to a change in guidelines in response to higher frequency of drug-resistant infections − Cost of drug administration (central lines, etc.) − Cost of nursing care − Cost of cohorting (including cost of leaving not unoccupied beds due to isolation of one patient restricting the use of the bed(s) in the same room) − Extended length of stay, whereby ICU and non-ICU days should be separated − Costs due to de-colonisation, if applicable, (e. g. mupirocin), re-testing, e.g. additional follow-up screening − Cost of non-standard surgical prophylaxis in colonised/infected patients, with more expensive drugs − Costs of infection prevention and control interventions as screening at hospital admission or before surgery
	Costs of long- term consequences of AMR infection	− Cost of additional laboratory tests or imaging to diagnose site of infection or foci of distant infectious metastatic foci − Cost of diagnosing and treating adverse events to 2nd, 3rd line etc. (Drugs used against MDROs infection need careful monitoring of toxicity and efficacy, thus more laboratory and radiological tests.) − Extra hospital admissions, or extra care for rehabilitation (e.g., respiratory, mobility, cognitive, neurological) and/or treatments required for disease sequelae directly linked to the drug-resistant infection, like recurrent infection, kidney failure, amputation, neurological sequelae, extra surgery
	Out-of-pocket expenditure borne by the patient for care	− Transport to and from the hospital (if the sole reason for the hospital admission was the infection) − Cost of funeral in cases of (attributable) death − Cost of (family/friend) care for the patient (e.g. hotel and meals to be near the hospital) due to excess length of stay of the patient related to the drug-resistant infection
	Surveillance and control activities^b^	− Costs of enhanced surveillance − Cost of any screening that is triggered − Costs of isolation, cohorting or contact precautions to the health care system, including facility design and operational costs
	Training of health care professionals and information/communication	− Costs of pre-service, in-service and continuous professional education per relevant cadre of human healthcare professional − Cost of any related public health or information campaign
	Legal and insurance costs (patient)	− Additional insurance costs to cover problems associated specifically with resistance − Litigation costs, when suing hospitals for transmission of resistance infection
	Legal and insurance costs (hospital)	− Litigation costs, when sued by patients for transmission of resistance infection − Costs of implementing or regulating and enforcing national robust, representative comprehensive surveillance programmes at all levels of health care from primary to tertiary levels
Indirect costs	Indirect patients’ costs: Loss of productivity/earning/opportunity when seeking treatment for the resistant infection (or colonisation) or dying from the resistant infection	− Value of foregone workdays value of foregone workdays because of disease sequelae related to the drug-resistant infection foregone treatments that depend on effectiveness of prophylaxis, like surgical interventions such as hip or knee replacements or caesarian sections − Foregone leisure time (NB: difficult to quantify) − Loss of productivity/earnings by family &visitors attending patient − Loss of caretaker (family/friend) productivity – (workdays foregone) − Psychological impact (factored in as QALY) − Other costs related to different life style (e.g. amputation leading to prosthesis or wheel chair; home renovation works to adapt to disability; nursing care costs, if unable to perform activities)
	Indirect hospital costs	− Reduced patient turnover and decreased revenues (due to longer hospital duration or to isolation/cohorting, or to decision not to perform a non-essential procedure –e.g. cosmetic surgery - etc.) − Reduced capacity of hospital (due to longer hospital duration or to isolation/cohorting − reputational costs borne by the hospital: any loss in hospital income related to the level of resistant infection/colonisation Note that a reduction in visits to one hospital may simply lead to an increase in visits for another. As this study takes a societal perspective only overall net reduction should be considered. (Assumption that no visits to the hospital are superfluous so that a reduction in visits due to fear of contracting a resistant pathogen imposes negative utility.)
	Societal/government	− Financial burden on the government for disability benefits
	Research and development of new antibiotics	− Cost to develop and bring a replacement drug to market^c^

^a^In sites where resistance is common and a greater percentage of fixed health care costs are spent managing it, the more the cost of overheads should be included in cost equations. Note that most colonization will not be treated with drugs, except cases like MRSA in patients awaiting surgery. However, colonization is likely to lead to more frequent visits, additional diagnostic tests, isolation of the patient, change in other contact precautions, etc.

^b^This work considers costs associated with phenotypic resistance in most cases. In the case of resistance surveillance, it considers genotypic resistance in that identification of resistance-carrying genes is assumed to impact on surveillance activities and screening in some cases

^c^When an antibiotic is rendered ineffective due to resistance, in a sense it is retired from the tool kit (in the language of accounting: it is fully depreciated). In companies or governments, reserves would have been set aside to account for the eventual need to replace the key asset. If this replacement cycle is done well, there is no downtime (for antibiotics, downtime is harm to patients from lack of effective therapy). So even if the antibiotic replacement cycle worked perfectly (with no harm to patients) there still is a cost: the effort to bring a replacement drug successfully to market. There is significant social waste since drug developers require many years (up to 15) from the university laboratory to an approved drug: it is difficult to judge the epidemiological need 15–-20 years onward. There currently are many ongoing patent races, with some duplication of effort as well

#### Cost burden of AMR on animals (Tables 6 and 7)

Direct costs related to the treatment of companion animals and animals in the food chain should be included. Ideally the effect on the quality of life of the owner of companion animals lost to resistant infections would also be considered. Indirect costs associated with resistance in food chain animals should be considered at the level of the individual farmer as well as at the sector-wide level if there is transmission that affects food chain supply (or if resistance in isolated cases decreases the overall demand for those food products). The different species of companion animals (dogs, cats, rabbits, birds, and others) and of food chain animals (ruminants, poultry, pigs, aquaculture, and others) should be considered individually.

**Table 6:** Costs related to a companion animal colonized or infected with a resistant pathogen (using its owner as proxy), necessary in a “One-Health” cost model

Data element	Category of costs	Individual costs
Direct	Costs of any treatment of the animal borne by the veterinary service (regardless of whether or not such costs are passed on to an insurance company.	− Cost of antibiotics − Cost of drug administration (central lines, etc) − Costs of diagnostic tests − Cost of nursing care − Cost of cohorting (including cost of leaving not occupied beds due to isolation of one patient restricting the use of the bed(s) in the same room) − Extended length of stay − Cost of non-standard surgical prophylaxis. Surgical prophylaxis in infected patients, with more expensive drugs − Extra hospital admissions, or extra care required for disease sequelae directly linked to the drug-resistant infection, like recurrent infection, kidney failure, amputation, neurological sequelae, extra surgery
	Out-of-pocket expenditure borne by the owner for care	− travel or transport to and from the veterinary clinic − special food, physiotherapy, transport − referring to specialists of complex cases − pet health insurance − Cost of disposal of remains/incineration/funeral
	Surveillance and control activities	− Costs of enhanced surveillance − Cost of any screening that is triggered − Costs of isolation, cohorting or contact precautions to the veterinary health care system − Costs for environmental decontamination of MDR bacteria
	Training of health care professionals and information/communication	− Costs of pre-service, in-service and continuous professional education per relevant cadre of veterinary healthcare professional − Cost of any related public health or information campaign
	Legal and insurance costs (patient)	− Additional insurance costs to cover problems associated specifically with resistance − Litigation costs, when suing hospitals for transmission of resistance infection
	Legal and insurance costs (hospital)	− Litigation costs, when sued by patients for transmission of resistance infection − Costs of implementing or regulating and enforcing national robust, representative comprehensive surveillance programmes among companion animals
Indirect	Loss of owner productivity when seeking treatment for the animal’s resistant infection (or colonisation) or when the animal dies from the resistant infection	− value of foregone workdays

**Table 7:** Costs related to farm animals colonised or infected with resistant pathogens (using farmers as proxy), necessary in a “One-Health” cost model

Data element	Category of costs	Individual costs
Direct	Costs related to resistant infection or colonisation with resistant bacteria within farm animals	− costs of 2nd, 3rd line antibiotic used for therapy vs growth promotion vs prophylaxis/metaphylaxis − Cost of veterinary consultation − Costs of diagnostic work-up − Reduction in farm productivity / output caused by AMR or antimicrobial restriction/ban
	Out-of-pocket expenditure by the farmer	− any related animal transport, slaughter − costs of culling animals − Restocking with animals/eggs
	Surveillance and control activities	− Costs of enhanced surveillance − Cost of any screening that is triggered − Costs of isolation, cohorting or contact precautions
	Legal and insurance costs	− Insurance costs − Litigation costs − Costs due to penalties or taxes associated with antimicrobial use; this is a reality in several EU countries (e.g. yellow card rule and special taxes on certain antimicrobial products in Denmark)
	Information and training costs	− Cost of any AMR public health or information campaign (e.g. including screening, biosecurity advising aimed at preventing or managing animals with resistant infection) − Costs of pre-service, in-service and continuous professional education per relevant cadre of veterinary healthcare professional and farm staff
Indirect costs	Loss of productivity to the farm or the wider food chain (if they are in some way dependent on output from the AMR affected farm and hence unable to maintain their normal level of productivity/sales).^a^	− Reduction in individual farm productivity / output − Longer time to market − Reduction in farm productivity / impact on the food chain (if food chain in some way dependent on output from the AMR affected farm and hence unable to maintain the normal level of productivity/sales) − Reduction in sales following a lower demand that is caused by knowledge of the existence of the resistant pathogen in the food chain
	Out-of-pocket expenditure by the consumer	− Increased cost of meat and other animal food products as a consequence of increased production costs

#### Cost burden of AMR on the environment (Table 8)

Environment here accounts for water, soil and air as well as plants and crops affected by environmental pollution with antibacterials and drug-resistant microorganisms from contaminated manure, pharmaceutical manufacturing waste, hospital waste, wastewater treatment facilities, untreated human waste, waste and runoff from aquaculture, livestock, and plant-based food production and processing facilities [20].

**Table 8:** Costs to the environment, necessary in a “One-Health” cost model

Data element	Category of costs	Individual costs
Direct costs	Cost of removing/decontaminating/cleaning/stemming flow	− Drug production effluent − Irrigation systems, farm run-off that contains resistant pathogens − Relevant waste in waste management systems − Relevant waste in drinking water storage and distributions systems
	Costs of surveillance and control programmes	− Costs of enhanced surveillance − Cost of any screening that is triggered − Cost of having to shift activities to non-contaminated areas − Cost to authorities of enforcing penalties on industries
	Training of food chain professionals (Environment)	− Costs of pre-service, in-service and continuous professional education per relevant cadre of environmental health professional
	Legal and insurance costs	− Cost to authorities of enforcing any penalties on industries − Cost to industry to comply with AMR-related regulations surrounding treatment, disposal, etc. − Costs of implementing or regulating and enforcing national environmental surveillance programmes on water, soil and air in different components of the One Health triad as appropriate
Indirect costs	Loss of productivity	− Overall economic loss in having unusable land while decontamination takes place (t.b.c.). Note this will be of greater significance in countries that are densely populated, densely apportioned economically (where the land is used to the maximum extent for economic purposes), rely on agriculture, and where water provision or flow is important economic asset.
	Loss to medical or non-medical trade and tourism from reduced trade/tourism (e.g. exclusion of a place as a tourist destination explicitly due to AMR-related concerns)	− Loss in income from local tourism due to resistance in swimming water, drinking water, any other contamination (reputational costs). − Loss to medical or non-medical trade and tourism from reduced trade/tourism (e.g. exclusion of a place as a tourist destination explicitly due to AMR-related concerns) − Loss to the travel industry due to cancellations or to longer, sustained reductions in travel

^a^All types of productivity loss to the farm should be put together (again, individual from societal cost should not be separated to avoid double counting. Rather the model will ultimately scale the effects of individual resistance up to where the more generalized societal costs come into play

While the costs associated with treating resistant infection do not generally apply to wildlife, the knock-on costs to species elimination, the undermining of ecosystems, and other related costs should be considered [21].

## Discussion

### The need for bottom-up costings of AMR

AMR is not a disease, with its own recognisable signs and symptoms and clearly associated health burden and epidemiological parameters. Rather it refers to species that have acquired drug resistance mechanisms, that have replicated, and overgrown other species that can colonize or infect people, animals, or the environment causing negative effects. The emergence of resistance is often due to selective pressure of antibiotic use, or during the acquisition of DNA from other species or strains, rendering the strain capable of resisting the inhibitory activity of some antibiotic (e.g. plasmid-mediated mechanisms of resistance). An infection that has not been a threat for many decades, over time can become resistant to currently available therapies, and potentially all existing therapies, and therefore become a threat again. This change within known existing pathogens, and the fact that one pathogen may be responsible for multiple types of infections (e.g. from UTI to CNS abscesses), can make AMR difficult to understand and address within the political sphere as well as within public discourse [2]. In addition, the slow emergence of the AMR problem makes it easily ignored in the present, and left to be addressed at a later time (which does not happen with rapidly spreading pathogens such as the SARS-CoV-2 virus, which has received immediate global attention and major resources).

Arguably this hinders to some extent our ability to place AMR appropriately within the hierarchy of political priorities and to address it with suitable urgency. An important tool in communicating concerns about AMR is to address the resulting economic consequences in a comprehensive and more immediately relevant way [9].

In addition, while previous rough, top-down estimates have helped ring the alarm bells within important international fora, they are insufficiently nuanced to help guide decisions. If a common, bottom-up framework such as this one can be applied in a selection of sites, we would be able to collect the necessary data to communicate the importance of AMR on the international and national agendas, including the appropriate focus on the various settings and sectors. Also, such cross-national estimates of costs within these settings and sectors can help guide research and development efforts, and appropriate funding schemes.

At national and local levels, a bottom-up costing is essential for choosing the optimal way to tackle AMR. Numerous different prevention, control, and treatment measures are available to help combat AMR. However, to compare existing measures or assess the potential of new ones we need to be able to estimate the costs that such interventions will impose as well as the costs that their implementation will off-set (in addition to estimates of their impact on the health burden). This bottom-up cost framework is intended to facilitate the estimation of these cost off-sets in particular, although the One Health cost “ingredients” will also cover most of the implementation costs associated with any intervention and thereby make any intervention-related costing exercise far simpler. Ultimately a bottom-up costing framework should help simplify any eventual economic analysis – whether it be cost-of-illness, cost-utility, or cost-benefit in structure.

### Challenges in isolating the costs of AMR

This work focusses on the additional cost of drug-resistant infections and drug-resistant colonisation, when compared to a drug-sensitive counterpart, answering the question “What is the impact of antibiotic-resistant infections (or colonization) relative to the same infection that would be susceptible to first line treatments?”. Sometimes, comparison to non-infected patients would also apply; this is particularly relevant when estimating the possible preventable burden of AMR, while focusing on an intervention that eliminates resistant as well as susceptible infections, like for example vaccination. In this scenario the question “What is the impact of antibiotic-resistant infections compared to a situation in which such infections would not occur at all, because they were prevented?” would be most relevant, which would require a comparison to no infection .

When it comes to the costs associated with mortality, the ability to attribute death to resistance is particularly challenging.^2^

For some of the components, especially at population level, attribution is fairly straightforward. For example, costs associated with enhanced resistance surveillance, consumption surveillance, public information campaigns to guide consumption, are fully and directly attributable to drug resistance. Other costs such as those associated with novel antibiotic R&D and antibiotic stewardship, infection control programs and associated training and implementation and monitoring costs, are also attributable to resistance. However, for most other components, especially at the individual level, attribution of cost to resistance is less clear. Methods to disentangle causation include subjective, labour-intensive chart review, or objective, costly cohort studies. In cohort studies, outcomes deriving from comparable patient groups with and without the drug-resistant infection/colonisation of interest are contrasted in order to measure the average additional cost imposed by the resistant form of the infection. Whether patients in the control groups should have a drug-susceptible infection, or should have no infection, is a subject of ongoing debate. Certain infections only happen because drug-resistance is present, like sepsis after an inappropriately treated urinary tract infection, or a surgical site infection after standard prophylaxis. In this case, a selection of control patients without infection is valid. For other infections this is much less clear. In certain cases the drug-resistant infection may just replace a drug-susceptible infection, and a selection of control patients with a drug-susceptible infection is most valid.

AMR costing studies will likely need to derive estimates of attributable costs from cohort studies such as those described above. Unfortunately, not many, high quality studies exist, and often their external validity is limited, leaving little precedent to utilize as a guide.

Furthermore, factors such as clinical manifestations – the distinction between colonisation and infection -- can add further layers of complexity when it comes to costs. For example, if the vague colonisation status of the patient is known, this is likely to increase costs: prophylaxis in the case of surgery or transplantation procedures would require a highly effective broader spectrum agent, which in many cases come with a higher price tag. Colonisation is also likely to lead to additional diagnostic tests, isolation of the patient, change in contact precautions (from standard precautions to standard plus contact precautions), and other costs. Conversely, while not knowing the colonisation status may lead to lower assumed costs, the costs associated with a transition to unanticipated resistant infection might be greater. The scenarios chosen to capture the different possible health states and their respective probabilities should reflect care realities at the local level (e.g. the degree to which active screening is performed, prophylaxis used, etc.).

The transmission of resistance within and between communities today increases the risk of being colonized and therefore further reduce the therapeutic options for future patients.

Finally, it should be noted that to comprehensively account for indirect costs may be particularly challenging, as highlighted in a recent framework developed to estimate the added value of new antibiotics in human health [22].

### Pathogen selection

For the purposes of this initial framework the starting point for selecting pathogen-drug pairs was the WHO list of priority pathogens for which new research is most urgently needed [23]. This was then expanded to include all possible AMR resistant drug-pathogen pairs believed to be most pressing for human, animal, and plant health, excluding (for the present time) non-bacterial pathogens. However, in practice, to maximize the usefulness of any costing exercise the list of relevant pathogen-drug combinations must be made at a local level.

Also, while the choice was made to focus this framework largely on bacterial microbes in the first instance, following the WHO Global Action Plan [2], it should be noted that the framework can be extended to fungal, viral, and parasitic diseases, where drug-resistance is becoming increasingly important.

Finally, although a species perspective was taken in this study, it is acknowledged that the microbiome, particularly those in the environment, include viable but not culturable (VNBC) bacteria that may also be reservoirs of resistance and resistance genes.

### Limitations

In focusing on bacteria, this work ignores costs imposed by resistance within other microorganisms. Even with regard to bacteria, the list is not exhaustive, as only major causes of disease/transmission/costs, were included. In some parts of the world the most worrying AMR will be amongst fungi, parasites, or viruses (e.g. *Plasmodium falciparum*, or HIV,) – none of which are explicitly listed in this work. In veterinary medicine anthelmintic resistance is an enormous problem already, whereas antibacterial resistance is probably not yet impacting on treatment of animal pathogens to the same extent as in humans. Resistance to antifungals also impose a non-negligible cost on healthcare services [24]. Recent examples include the global spread of *Candida auris* infection and the azole-resistant *Aspergillus fumigatus* [25, 26]. We hope to address the costs imposed by these pathogens in future work.

Finally, in trying to create a framework that can be used by researchers or government worldwide to estimate the cost of AMR, this work is likely to miss some important details in how and where AMR imposes costs locally. Local studies may be needed to adapt the framework to clinical norms and the epidemiological reality to effectively capture costs.

A further challenge is the lack of research capacity and funding, especially - but not only - in LMICs; the quality and accuracy of the data necessary to obtain reliable cost estimates is unlikely to be readily available in most countries.

## Conclusion and future developments

Attaining a realistic understanding of how and to what extent antibiotic resistance affects society is a challenging task. We hope that this work helps to pave the way to a clearer view of AMR costs and ultimately helps inform important decisions across the interconnected domains of human, animal, and environmental health in the years to come. Whether these decisions concern potential infection control interventions, targeting of surveillance efforts, how best to steer research and development efforts, or exciting innovative new ways of tackling AMR, a credible and nuanced assessment of AMR-related costs is essential. Using a sufficiently granular, bottom-up framework across multiple sites we should be able to achieve the necessary global estimates needed to support major international initiatives and better guide major R&D funding, while remaining sufficiently flexible to adapt to local realities and guide resource allocation.

## Data Availability

Not applicable.

## Footnotes

1 In some cases, comparison to a non-infected population is more relevant, in particular where there have been certain types of preventive measures. For example, if vaccination prevents all future drug-resistant and susceptible infections, the harm of drug-resistance as well as infections in general is eliminated, and therefore the most appropriate comparison would calculate the cost impact of resistant infections relative to no infection..

2 This challenge has been repeatedly brought to the fore in the litigation of damage claims after death following resistant infection.
